# In vivo assessment of optical properties of melanocytic skin lesions and differentiation of melanoma from non-malignant lesions by high-definition optical coherence tomography

**DOI:** 10.1007/s00403-015-1608-5

**Published:** 2015-11-13

**Authors:** M. A. L. M. Boone, M. Suppa, F. Dhaenens, M. Miyamoto, A. Marneffe, G. B. E. Jemec, V. Del Marmol, R. Nebosis

**Affiliations:** Department of Dermatology, Hôpital Erasme, Université Libre de Bruxelles, Route de Lennik 808, 1070 Brussels, Belgium; Research Department, Agfa HealthCare, Mortsel, Belgium; Department of Dermatology, Roskilde Hospital, University of Copenhagen, Roskilde, Denmark; Research Department, Agfa HealthCare, Munich, Germany

**Keywords:** High-definition optical coherence tomography, Benign nevi, Dysplastic nevi, Melanoma, Melanocytic lesions, In vivo optical properties, Scattering, Absorption, Light attenuation, Reflectance

## Abstract

One of the most challenging problems in clinical dermatology is the early detection of melanoma. Reflectance confocal microscopy (RCM) is an added tool to dermoscopy improving considerably diagnostic accuracy. However, diagnosis strongly depends on the experience of physicians. High-definition optical coherence tomography (HD-OCT) appears to offer additional structural and cellular information on melanocytic lesions complementary to that of RCM. However, the diagnostic potential of HD-OCT seems to be not high enough for ruling out the diagnosis of melanoma if based on morphology analysis. The aim of this paper is first to quantify in vivo optical properties such as light attenuation in melanocytic lesions by HD-OCT. The second objective is to determine the best critical value of these optical properties for melanoma diagnosis. The technique of semi-log plot whereby an exponential function becomes a straight line has been implemented on HD-OCT signals coming from four successive skin layers (epidermis, upper papillary dermis, deeper papillary dermis and superficial reticular dermis). This permitted the HD-OCT in vivo measurement of skin entrance signal (SES), relative attenuation factor normalized for the skin entrance signal (*µ*_raf1_) and half value layer (*z*_1/2_). The diagnostic accuracy of HD-OCT for melanoma detection based on the optical properties, *µ*_raf1_*, SES* and *z*_1/2_ was high (95.6, 82.2 and 88.9 %, respectively). High negative predictive values could be found for these optical properties (96.7, 89.3 and 96.3 %, respectively) compared to morphologic assessment alone (89.9 %), reducing the risk of mistreating a malignant lesion to a more acceptable level (3.3 % instead of 11.1 %). HD-OCT seems to enable the combination of in vivo morphological analysis of cellular and 3-D micro-architectural structures with in vivo analysis of optical properties of tissue scatterers in melanocytic lesions. In vivo HD-OCT analysis of optical properties permits melanoma diagnosis with higher accuracy than in vivo HD-OCT analysis of morphology alone.

## Introduction

One of the most challenging problems in clinical dermatology is the early detection of melanoma [4, 42, 46]. Since clinical diagnosis may be difficult, non-invasive imaging techniques have been developed to enhance early diagnosis in challenging cases [10, 17]. Dermoscopy and reflectance confocal microscopy (RCM) are both able to considerably improve the diagnostic accuracy for melanoma, especially when used synergistically [11, 32, 33, 48]. However, diagnosis strongly depends on the experience of physicians [2, 31].

New diagnostic tools providing automated classification of pigmented skin lesions usable by non-experts have been proposed [23]. Spectral methods fall into this class of emerging new techniques holding the promise to provide quantitative criteria for melanoma diagnosis and to improve early diagnosis [37]. Multispectral information can be assessed both in the spatial domain (multispectral digital dermoscopy [22]) or in the frequency domain (spectroscopic methods such as diffuse-reflectance spectroscopy [21, 41, 54], Raman spectroscopy [36, 52] and fluorescence spectroscopy [15, 34]).

These methods evaluate the colour of a lesion by measuring its reflectance as a function of the wavelength. A comprehensive analysis of pigmented lesions under both ultraviolet and infrared radiations has previously been described in 1976 [37]. It was stated that infrared photographs tends to discriminate the different types of melanocytic lesions, with melanoma showing a relatively high degree of correlation with low infrared reflectance [37].

RCM and optical coherence tomography (OCT) are both technologies operating in the near-infrared (NIR) optical window. Conventional OCT enables the non-invasive imaging of structures with a non-cellular resolution (7.5–15 µm) up to a depth of about 1 mm, while high-definition OCT (HD-OCT) provides cellular (3 µm) resolution up to a depth of 570 µm [3, 5, 6, 8, 18, 19, 25, 44]. Conventional OCT has been used for non-invasive tumour thickness measurement in melanocytic skin lesions. A variable correlation with histopathology could be observed depending on OCT technology used [24, 38]. HD-OCT offers additional structural and cellular information on melanocytic lesions complementary to that of RCM [9]. However, the diagnostic potential of HD-OCT seems to be not high enough for ruling out the diagnosis of melanoma if based on morphology analysis [20].

In a recent study, it was demonstrated that HD-OCT permits to quantify the reflectance of NIR light in a volume of skin as function of depth [7]. Therefore, the aim of this paper was to quantify in vivo optical properties such as light attenuation in melanocytic lesions by HD-OCT. An additional objective was to determine the best critical value of these optical properties for melanoma diagnosis.

## Methods

### Study sample

The cases for this retrospective pilot study were retrieved from patient files collected at first author’s private practice between 2012 and 2015. The cases contain melanocytic lesions including benign nevi (BN), dysplastic nevi (DN) and melanomas (MM). The inclusion criteria were: (i) selection of clinically suspected melanocytic lesions for full excision based on dermoscopic or combined dermoscopic/RCM evaluation and subsequent histopathologic diagnosis and (ii) availability of relevant (see procedure) HD-OCT images taken prior to excision.

Approval from local ethical committee was obtained (P2015/301). All patients provided informed consent for imaging their lesion by HD-OCT (Skintell^®^, Agfa Healthcare, Mortsel, Belgium). We conformed to the Helsinki Declaration with respect to human subjects in biomedical research. All international rules governing clinical investigation of human subjects were strictly followed. This study affected neither the routine diagnosis nor the treatment of the lesions presented by the included subjects.

### Histopathology

Histopathologic analysis was carried out by two board-certified histopathologists who classified each lesion according to histopathological criteria for melanocytic lesions described elsewhere [12–14].

### High-definition optical coherence tomography

Instruments and acquisition methods and technical details have been previously described [5, 9]. For the purpose of this study we will remind what follows.

HD-OCT has four basic design principles: (i) a time domain OCT providing depth information by the position of the reference mirror, (ii) full-field illumination by a light source enabling very low lateral coherence and permitting a high-speed 3-D sharp image acquisition, (iii) high-power tungsten lamp with Gaussian filter and ultrahigh bandwidth (1300 ± 100 nm). This permits high-depth resolution of 3 µm and (iv) full-field domain OCT system with dynamic focus tracking: the focal plane is continuously moved through the skin sample. This ensures a high lateral resolution over the entire depth of 3 µm. HD-OCT offers a constant homogeneous resolution of 3 µm in all three dimensions. The system is capable of capturing a cross-sectional and en face image in real time, as well as of fast real time 3-D acquisition. A sharp image at all depth is guaranteed up to a theoretical depth of 570 µm. The field of view is 1.8 × 1.5 mm. The total light power at the tissue is <3.5 mW.

### HD-OCT focus tracking

Skin is a highly turbid medium. Light which propagates through skin is scattered and absorbed resulting in attenuation of this light. In contradiction to a “normal” light detector HD-OCT is only detecting photons which reach the detector on an almost straight path—the ballistic portion of the photons or photons which were only forward scattered a few times [49].

The resulting attenuation coefficient was estimated by Jacques et al. [27–29]. Since absorption is much smaller than scattering for tissues in the near-infrared spectrum, absorption can be neglected [29]. HD-OCT operates in the second diagnostic window of the NIR spectrum [47] and hence the main attenuation mechanism for HD-OCT is scattering. Measuring tissue optical properties in vivo is only applicable to OCT when operating focus tracking mode which is the case for HD-OCT [27]. Table 1 provides definitions and abbreviations of optical properties adapted from the literature [27, 29, 30, 35, 49, 50].

**Table 1:** Definition of optical properties adapted from the literature [33, 35, 36, 38–40]

Optical properties	Dimension	Definition
Lambert–Beer expression	Dimensionless	$\frac{I(z)}{{I_{\text{o}} }} = e^{ - \mu z}$ Light intensity *I*(*z*) after a path length *z* in medium with homogenous absorber without scattering. Permits calculation of attenuation coefficient
OCT signal	Dimensionless	Light intensity which is leaving the skin. First light penetrates into the skin and is attenuated on the path down—than light is backscattered/reflected at objects and travels back up to the surface (on the way up it is again attenuated). Finally the light leaves the skin. OCT is only detecting photons which reach the detector on an almost straight path—the ballistic portion of the photons
Jacques expression	Dimensionless	$R\left( z \right) = \rho e^{ - \mu z}$ Intensity of an OCT signal depends on the attenuation *µ* of light while penetrating and leaving the skin and on the backscatter strength *ρ* of the imaged object
Anisotropy of scatter *g* (0–1)	Dimensionless	Characterizes tissue scattering in terms of the relative forward versus backward direction of scatter. It determines the effectiveness of scattering after a single scattering event. Hence, it represents a measure of the amount of forward direction retained after a single scattering event
*a*(*g*) if *g* 0.8 → 1, then an important (85 %) reduction in scattering is noticed.	Dimensionless	Describes the influence of anisotropy of scatter on light attenuation. Drops from 1 to 0 as g increases from 0 to 1. The factor *a*(*g*) has only a significant influence on attenuation for *g* values >0.8 which are typical for skin. The larger the scattering structures the larger *g* and the lower the tissue attenuation
*G*(*g*,NA)	Dimensionless	Optical geometrical factor. Equals 1.1 for HD-OCT and describes the average photon path length Depends on the numerical aperture (NA) of the lens and the anisotropy of scatter *g* (see further)
Local backscatter strength *ρ* if *g* 0.8 → 1, then only a small reduction (8 %) in backscattering is observed.	Dimensionless	$\rho = \mu s Lf b(g,NA)$ Whereby Lf corresponds to the coherence length of the OCT and is for HD-OCT 3 µm; *b*(*g*,NA) is the fraction (1 to 0) of scattered light that backscatters into the lens for detection. The higher the anisotropy of scatter g the lower *b*(*g*,NA)
Total attenuation coefficient	µm^−1^	*µ* = (*µ*_s_ *a*(*g*) + *µ*_a_) 2 *G*(*g*,NA) if light source ≪1300 nm *µ* = *µ*_s_ *a*(*g*) 2 *G*(*g*,NA) if *µ*_a_ ≪ *µ*_s_ at 1300 nm
Absorption coefficient *µ*_*a*_	µm^−1^	The cross-sectional area (cm^2^) for absorption per unit of volume of medium (cm^3^) containing a uniform distribution of identical absorbers
Scattering coefficient *µ*_s_	µm^−1^	Describes the number of scattering events per µm in a medium containing a uniform distribution of identical scatterers at a concentration defined as a volume density ν_s_
Reduced scattering coefficient *µ*′_s_	µm^−1^	Is related to the scattering coefficient as follows: *µ*′_s_ = *µ*_s_ (1 − *g*). This is the pertinent term for describing light transport when multiple scattering occurs as the anisotropy of scatter is taken into account
Mean free path mfp	µm	The inverse of scattering coefficient. In the diffusion (scattering) process, the photons moving through the skin do so in a series of steps of random length and direction (i.e. random walk). Each step begins with a scattering event and is equally likely to be taken in any direction and ends with a next scattering event
Reduced mean free path mfp		The inverse of the reduced scattering coefficient. Distance between two isotropic scattering events
Tissue half value thickness *z*_1/2_	µm	Depth value under the skin surface corresponding with half of the backscattering intensity measured at stratum corneum
Rayleigh scattering (Rayleigh limit)		Scattering due to tissue structures that are considerably smaller than the light wavelength. This type of scattering occurs in all directions (isotropic scattering) but is peaked equally in the forward and backward directions. This scattering is also wavelength dependent (varies as the inverse fourth power of the wavelength)
Mie scattering (Mie regime)		Scattering due to larger spherical particles with diameters on the order of light wavelengths or larger. The scattering takes place mainly in the forward direction (anisotropic scattering) This scattering is less wavelength dependent (varies as 1/*λ* ^*b*^ with *b* ≥ 1)

### Procedure (Figs. 1, 2, 3)

The selection of spots in melanocytic lesions imaged by HD-OCT was based on dermoscopic or combined dermoscopic/RCM evaluation. Subsequently the selected spots were marked with a plastic ring of 2 mm diameter. A 3-D HD-OCT DICOM (digital imaging and communication in medicine) image taken at the centre of this ring was selected. This DICOM file was then opened using ImageJ^®^ software. A single square region of interest (ROI) in the en face view (green square) was selected in each melanocytic lesion based on the presence of relevant morphologic features as described in previous papers [9, 20]. To exclude obvious “artefacts” such as air bubbles or skin furrows, a square ROI was carefully chosen. The size of the ROI was set at least to 300 × 300 µm.

**Fig. 1:**
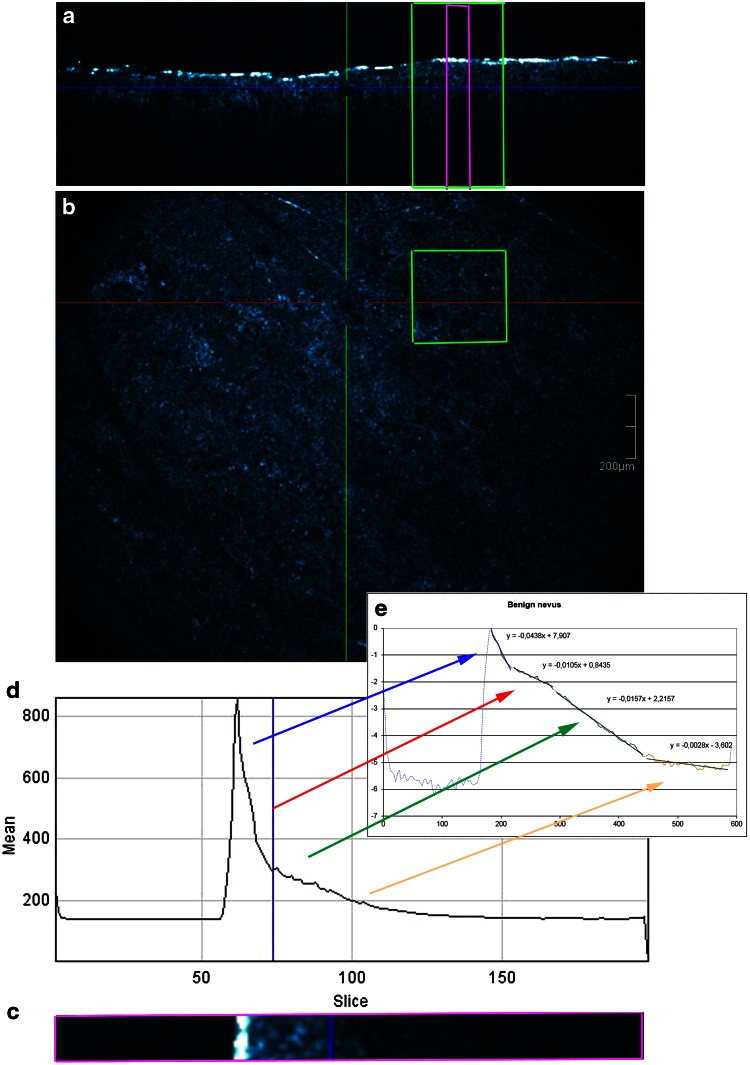
Benign nevus. **a**, **b** A 3-D HD-OCT DICOM (digital imaging and communication in medicine) image of each melanocytic lesion is selected. To exclude artefacts such as skin furrows or air bubbles, a square region of interest is chosen in the en face image based on the presence of relevant morphologic features of melanocytic lesions (*green square*). A plot *z*-axis profile of the scanned volume is performed. **c**, **d** The obtained graph displays the reflectance of the ballistic photons [OCT signal: measured on the *y*-axis with arbitral units (AU)] versus imaging depth which is indicated on the *x*-axis (slice numbers from 0 to 200 axial position of depth). The slice numbers are multiplied by factor 3 (slice thickness equals 3 µm) to correspond to the real depth in micron (from 0 to 600 µm: see *x*-axis in (**e**). **e** Semi-log plot: an exponential function becomes a *straight line* given by *y* = *ax* + *b.* Four successive layers (epidermis, upper papillary dermis, deeper papillary dermis and superficial reticular dermis) with clear exponential decay are identified and plotted. A *straight line* is fitted in each of the four layers (*i* = 1–4) resulting in equation of the type *y*_*i*_ = *ax*_*i*_ + *b* whereby *a* is proportional to the attenuation coefficient for each of the four layers

**Fig. 2:**
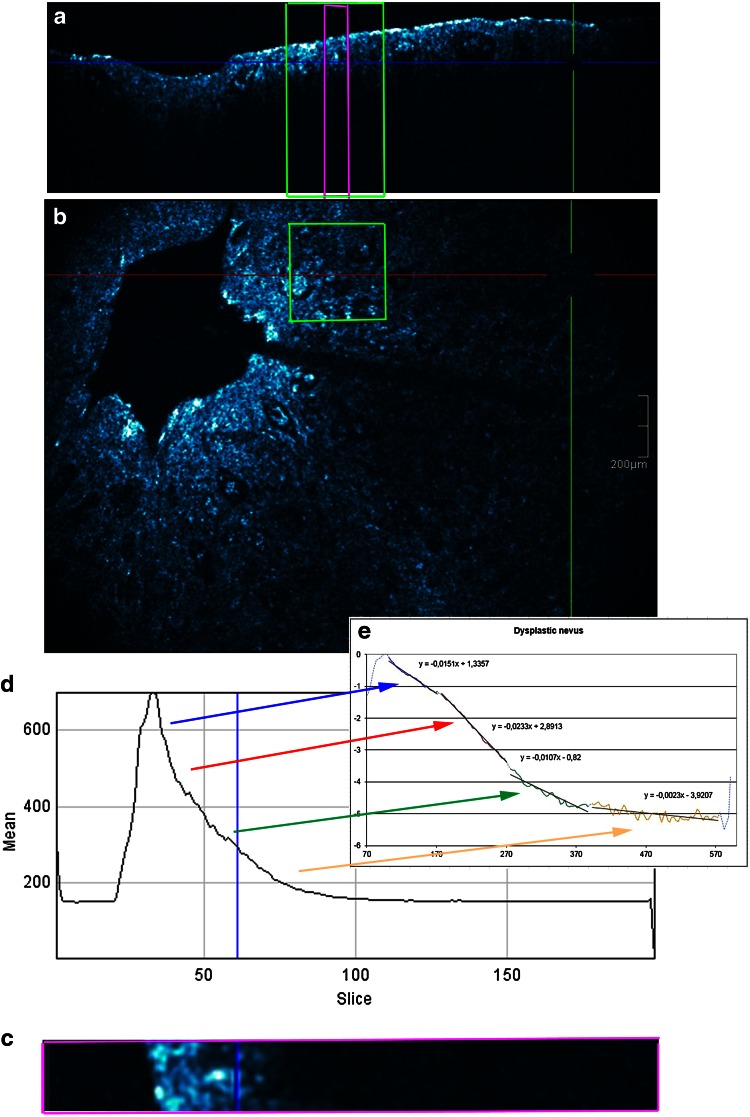
Dysplastic nevus. **a** Cross-sectional image, **b** en face image, **c** section of the cross-sectional image, **d** graph displaying reflectance versus depth of focus and **e** semi-log plot

**Fig. 3:**
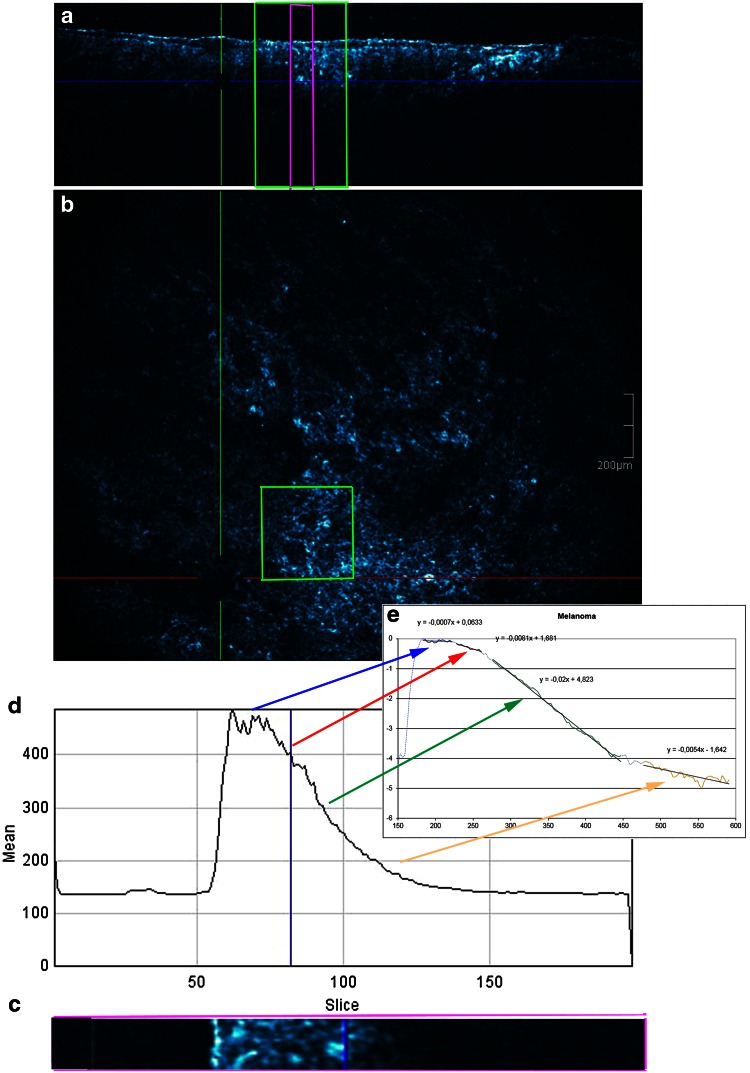
Malignant melanoma. **a** Cross-sectional image, **b** en face image, **c** section of the cross-sectional image, **d** graph displaying reflectance versus depth of focus and **e** semi-log plot

The obtained graph displayed the reflectance and attenuation of the ballistic photons [OCT signal: measured on the *y*-axis with arbitral units (AU)] versus imaging depth which was indicated on the *x*-axis (from 0 to 200 axial position of depth). In a next step, the offset corresponding to the mean signal within gel was removed from each OCT signal. The first peak corresponded to the skin entrance signal (SES). All the HD-OCT values will be divided by SES to normalize them to the SES. The natural logarithm ln(*x*) of those values will be taken. The slice numbers needed to be multiplied by factor 3 to correspond to the real depth in micron (from 0 to 600 µm).

The decay with imaging depth of reflectance (backscattered intensity) of ballistic photons is a process that can be well modelled by an exponential relationship [26, 29].

When operating in the NIR diagnostic window, absorption becomes negligible and the slope of the exponential attenuation is proportional to the reduced scattering coefficient *µ*′_s_. Analysis by semi-log plot (an exponential function becomes a straight line given by *y* = *ax* + *b*) of the exponential profile of light attenuation, can provide information on tissue scattering properties [26, 29].

Four successive layers (epidermis, upper papillary dermis, deeper papillary dermis and superficial reticular dermis) with clear exponential decay were identified and plotted. A straight line was fitted in each of the four layers (*i* = 1–4) resulting in equation of the type *y*_*i*_ = *ax*_*i*_ + *b* whereby a was proportional to the attenuation coefficient for each of the four layers given by *µ* = a/2.

Three optical properties were measured:

- Relative attenuation factor normalized to skin entrance signal for each of the four layers (*µ*_raf1-4_; µm^−1^)
- Skin entrance signal (SES; arbitrary unit)
- *z* value under the skin surface at which the OCT signal equals half of the SES, half value layer (*z*_1/2_; µm)

### Statistical analysis

One-way analysis of variance (ANOVA) was used to compare means of three samples (BN,DN and MM) using the F distribution. Prior to the Anova test, Levene’s Test for “Equality of Variances” was performed. If the Levene’s test was positive (*p* < 0.05) then the variances in the different groups were different (the groups are not homogeneous) and a logarithmic transformation to the data has been performed. Moreover, Scheffé test was used for all pairwise comparisons.

The best critical value of all HD-OCT assessed optical properties was determined by applying the receiver operating characteristic curves (ROC curves). This is a graph displaying the relationship between the true-positive rate (on the vertical axis) and the false-positive rate (on the horizontal axis) (Table 2).

**Table 2:** In vivo HD-OCT measured values of optical properties of melanocytic lesions according to thee subgroups

Subject #	Relative attenuation factor *µ*_raf1_	Skin entrance signal SES	Half value layer *z*_1/2_
*Benign nevi*			
1	0.0437	406	7.07
2	0.0664	817	2.79
3	0.0248	505	8.18
4	0.0780	818	1.89
5	0.0593	847	1.84
6	0.0388	1101	3.03
7	0.0152	683	5.22
8	0.0766	1066	2.02
9	0.0520	935	6.86
10	0.0220	760	5.42
11	0.0502	889	3.31
12	0.0454	731	7.88
13	0.0254	515	6.21
14	0.0388	678	6.64
15	0.0438	723	3.39
*Dysplastic nevi*			
1	0.0160	475	4.84
2	0.0357	721	3.16
3	0.0407	716	4.80
4	0.0158	549	6.05
5	0.0166	731	6.24
6	0.0168	955	6.10
7	0.0161	1116	4.92
8	0.0067	733	12.43
9	0.0076	723	9.28
10	0.0214	258	7.11
11	0.0186	227	7.7
12	0.0237	678	4.73
13	0.0249	309	6.73
14	0.0076	234	10.31
15	0.0101	474	9.21
*Melanoma*			
1	0.0164	562	7.16
2	0.0021	490	9.4
3	0.0067	432	11.62
4	0.0033	154	22.98
5	0.0008	286	11.22
6	−0.0012*	228	18.12
7	0.0002	570	28.05
8	0.0047	362	24.39
9	−0.0032	421	8.8
10	0.0007	351	12.79
11	−0.0050	400	12.52
12	0.0004	457	9.69
13	0.0002	196	10.72
14	−0.0024	285	14.71
15	−0.0025	253	14.4

* Negative values means increasing back scatter strength with increasing depth

Based on these critical values absolute and relative frequencies were calculated for the three groups. Chi-squared (χ^2^) test was employed to compare each melanocytic group versus the other melanocytic groups. The phi (*φ*) coefficient, employed to weight diagnostic power of each significant parameter, is a measure of association of two binary variables and is related to the Chi-squared (χ^2^) statistic by the formula*: φ*^2^ = χ^2^/*n,* where *n* equals the total number of observations.

Calculations were made using MedCalc^®^ statistical software version 14.12.0.

## Results

### Subjects

We included in this retrospective study 45 cases. These cases were retrieved from 2249 patient files collected between 2012 and 2015. Based on dermoscopic or dermoscopic/RCM assessment, 321 cases underwent full excision for histopathologic diagnosis. Forty-nine of these cases have been imaged by HD-OCT whereof 45 cases were retained based on the presence of relevant morphologic features of melanocytic lesions as described previously [9, 20]. The cases comprise 15 BN, 15 DN and 15 melanomas. All melanomas were histopathologically classified as superficial spreading melanomas with a Breslow-index varying between 0.31 and 0.72. The lesions belonged to 25 females and 20 males with skin type I–III and ages ranging from 25 to 70 years (median 51 years).

### Quantitative in vivo evaluation (Table 3)

**Table 3:** Quantitative evaluation of optical properties of melanocytic lesions

Optical properties	Malignant melanoma	Dysplastic nevus	Benign nevus
Relative attenuation factor of first layer *µ*_1_ (µm^−1^)	0.0014 [±0.0026] (*p* < 0.001)*	0.0186 [±0.005] (*p* < 0.001)	0.04541 [±0.0097] (*p* < 0.001)
Skin entrance signal (arbitrary units)	363 [±65] (*p* < *0.001*)	593 [±134]	765 [±99]
Half value layer (*z*_1/2_) (µm)	13.36 [±3.15] (*p* < 0.001)	6.50 [±1.26] (*p* < 0.001)	4.22 [±1.15] (*p* < 0.001)

* *p* values are mentioned whenever appropriate; for details see “Results” section

1. *µ*_raf1_: a significant difference (*p* < 0.001) could be observed between the three subgroups in the upper layer. *µ*_raf1_ decreased progressively from benign to malignant lesions. No significant differences could be demonstrated between the three groups for the other layers *µ*_raf2-4_. (Fig. 4).

**Fig. 4:**
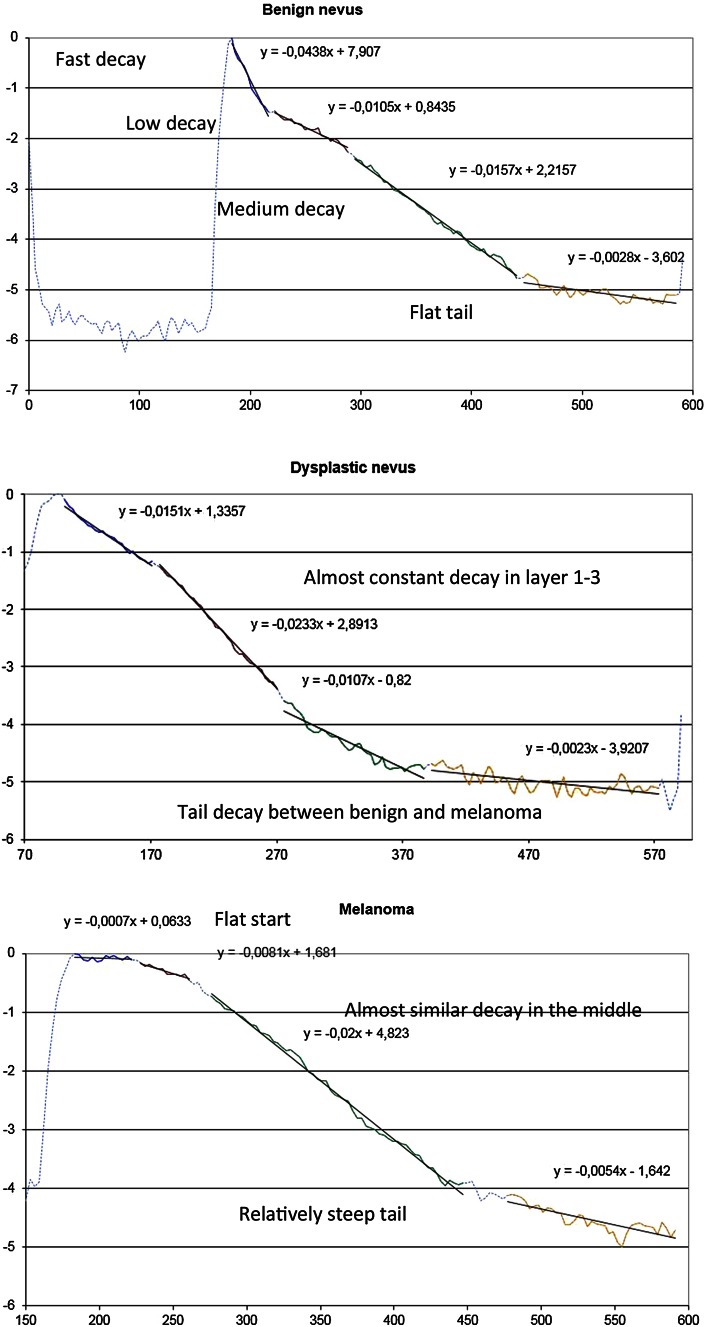
Comparison of trend lines of relative attenuation factor at different skin layers in benign nevus (*upper part*), dysplastic nevus (*middle part*) and melanoma (*lower part*)
2. SES: a significant (*p* < 0.001) reduction in skin entrance signal could be observed in the melanoma group compared to the two other groups.
3. *z*_1/2_: a significant difference (*p* < 0.001) could be observed between the three subgroups. This value increased progressively with malignancy.

### Best critical value of optical properties

Critical values permitting differentiation by HD-OCT of the three subgroups could be defined by applying the receiver operating characteristic curves (ROC curves) and are displayed in Table 4.

**Table 4:** Selection of best critical value of optical properties of melanocytic lesions by applying receiver operating characteristic curves

Optical properties of melanocytic lesions	Critical value	Area under ROC curve	Sensitivity (%)	Specificity	Accuracy	Positive likelihood ratio
Differentiating malignant melanoma						
Relative attenuation coefficient first layer	<0.0067/µm	0.981	93.3	96.7	95.60	28
Skin entrance signal	<475	0.86	80.0	83.3	82.2	4.8
Tissue half value thickness	>8.18 µm	0.960	93.33	86.67	88.9	7
Differentiating benign nevi						
Relative attenuation coefficient first layer	>0.0248/µm	0.847	80.0	90.0	88.89	8
Skin entrance signal	>678	0.832	80.0	76.6	77.78	3.14
Half value layer	<6.64 µm	0.847	73.3	73.3	73.33	2.75

### Absolute and relative frequencies of optical properties (Table 5)

**Table 5:** Absolute and relative frequencies of optical properties of melanocytic lesions in relation to critical values as assessed in vivo by high-definition optical coherence tomography

	Malignant melanoma (*N* = 15)	Dysplastic nevi (*N* = 15)	Benign nevi (*N* = 15)
Differentiating melanoma			
Relative attenuation coefficient at first layer *µ* < 0.0067/µm	14 (93.3 %) (*p* < *0.001*)*	1 (6.6 %)	0 (0.0 %)
Skin entrance signal <475 a.u.	12 (80.0 %) (*p* < 0.001)	4 (26.6 %)	1 (6.6 %)
Half value layer *z*_1/2_ > 8.18 µm	14 (93.3 %) (*p* < 0.001)	4 (26.6 %)	0 (0.00 %)
Differentiating benign nevi			
Relative attenuation coefficient at first layer *µ* > 0.0248/µm	0 (0.0 %)	3 (20.0 %)	13 (86.6 %) (*p* < 0.001)
Skin entrance signal > 678 a.u.	0 (0.0 %)	7 (46.6 %)	12 (80.0 %) (*p* > 0.01)
Half value layer *z*_1/2_ < 6.64 µm	0 (0.0 %)	8 (33.3 %)	10 (66.6 %) (*p* < 0.01)

* *p* values have been added whenever appropriate; for details see “Results” section

1. Differentiation of MM from non-malignant lesions:
  *µ*_raf1_ < 0.0067/µm *was* a high sensitive (SS) and specific (SP) feature of MM group (93.3 and 96.7 %, respectively; *ϕ* = 0.90 and *χ*^2^ = 36.45, *p* < 0.001). Moreover, the negative predictive value (NPV) was 96.7 % and the positive predictive value (PPV) was 93.3 %. The diagnostic accuracy was 95.6 %.
  SES < 475 a.u. was a sensitive and specific feature of MM group (80.0 and 83.3 %, respectively, NPV = 89.3 %, PPV = 70.6 %, *ϕ* = 0.62 and *χ*^2^ = 17.06, *p* < 0.001).
  *z*_1/2_ > 8.18 µm was a high sensitive and specific feature of MM group (93.3 and 86.7 %, respectively, NPV = 96.3 %, PPV = 77.8 %, *ϕ* = 0.77 and *χ*^2^ = 26.67, *p* < 0.001).
2. Differentiation of benign nevi from non-benign nevi (DN and MM)
  *µ*_raf1_ > 0.0248/µm was a sensitive and high specific feature of the BN group (80.0 and 90.0 %, respectively, NPV = 93.0 %, PPV = 81.0 %, *ϕ* = 0.60, *χ*^2^ = 16.25, *p* < 0.001). Values higher than this cutoff value were absent in all lesions of the MM group.
  SES > 678 a.u. was moderate sensitive and specific for the BN group (80.0 and 76.7 %, respectively, NPV = 88.0 % and PPV = 63.0 %, *ϕ* = 0.54, *χ*^2^ = 13.16, *p* < 0.01). In MM no value higher than 678 a.u. could be observed.
  *z*_1/2_ < 6.64 µm was a moderate sensitive and specific feature of the BN group (both 73.3 %, NPV = 85.0 % and PPV = 58.0 %, *ϕ* = 0.45 and *χ*^2^ = 8.93, *p* < 0.01). Values lower than this cutoff value were absent in all lesions of the MMgroup.

## Discussion

Non-invasive imaging techniques have been introduced to improve the early detection of melanoma which can be often challenging with the naked eye alone [10, 17]. Dermoscopy and RCM were shown to improve diagnostic accuracy in this field [11, 32, 33, 48]. In a recent study, almost no melanomas were misclassified and consequently undertreated when both techniques were used in combination: sensitivity 97.82 %, specificity 92.44 %, PPV 87.37 % and NPV 98.75 % [1]. Moreover, a significant reduction in numbers needed to excise could be determined by combining both devices [1, 43]. The problem with dermoscopy and RCM, however, is that their diagnostic performances varies with the user’s experience and would be consequently poor for non-expert physicians [2, 31]. A user-independent, automated classification of pigmented skin lesions based on their optical properties could be achieved by other techniques, such as spectrophotometric technologies [23]. The diagnostic potential of HD-OCT is traditionally thought to be insufficient for ruling out the diagnosis of melanoma, due to limitations of its cellular resolution, as compared to RCM [20]. However, it was shown that HD-OCT allows quantifying the skin reflectance as function of depth [7]. The present study is—to our knowledge—the first one describing the optical properties of benign and malignant melanocytic skin lesions assessed in vivo by means of HD-OCT. We assessed the scattering properties of 45 melanocytic lesions, including 15 benign nevi, 15 dysplastic nevi and 15 melanomas.

Only ballistic photons backscattered to the detection system of the HD-OCT contribute to the image; the decay with depth of focus of reflectance of ballistic photons is a process that can be well modelled by an exponential relationship [26, 29]. The technique of semi-log plot whereby an exponential function becomes a straight line given by *y* = *ax* + *b* has been implemented on HD-OCT signals coming from four successive skin layers (epidermis, upper papillary dermis, deeper papillary dermis and superficial reticular dermis). This permitted the HD-OCT in vivo measurement of skin entrance signal (*SES*), relative attenuation factor normalized for the skin entrance signal (*µ*_raf1_) and half value layer (*z*_1/2_).

A highly significant difference between each melanocytic group could be observed with regard to the measured optical properties. A reduction in *µ*_raf1_ and *SES* and increase of *z*_1/2_ could be noticed with increasing malignancy. Spectrophotometric studies provided evidence of decreased reflectance with increased malignancy of melanocytic lesions [37]. From a clinical point of view, it is more disastrous if MM is misdiagnosed as BN then if a DN is mistaken for a MM. Hence, decision criteria minimizing the false-negative rate deserve priority. Therefore, critical values could be determined for these optical properties permitting differentiation of malignant melanoma from non-malignant melanocytic lesions with high area under the roc curve (AUC) values (0.98, 0.86 and 0.96, respectively) and high positive likelihood ratio’s (28, 4.8 and 7, respectively).

The diagnostic performance of HD-OCT in discriminating MM from non-melanoma based on 3-D cellular and micro-architectural morphological features has been found to be moderate with an NPV (Negative Predictive Value) of 89.7 % [20]. High false-negative rates were observed in very thin MM and high false-positive rates in DN [20]. The present study dealt with superficial spreading melanomas and provided evidence that the diagnostic accuracy of HD-OCT based on the optical properties, *µ*_raf1_*, SES* and *z*_1/2_ scored much better (95.6, 82.2 and 88.9 %, respectively). High NPV could be found for these optical properties (96.7, 89.3 and 96.3 %, respectively) reducing the risk of mistreating a malignant lesion to a more acceptable level (3.3–7.3 % instead of 11.3 %). The role of spectrophotometry (SPT) in early diagnosis of melanoma in 10 studies has been compared [2]. The authors concluded that low diagnostic accuracy with moderate NPV represented the main hamper for the introduction of SPT technology in clinical practice. HD-OCT enables the rather unique combination of in vivo morphological analysis of cellular and micro-architectural structures with in vivo analysis of optical properties of tissue scatterers.

Optical property measurements sensitive for scattering and anisotropy factor ***g*** offer the best means of characterizing the micro-architecture of cells and tissues in general and melanocytic lesions more specifically [27, 45, 47, 49, 55]. Although a lot of variation in data exists, in general there appears to be a trend toward increasing *g* and decreasing reduced scattering coefficient *µ*′_s_ as the wavelength increases. In normal skin with photo type I-III, at 1300 nm (second NIR optical window) the mean value for reduced scattering coefficient *µ*′_s_ is 0.001 (±0.0005) [53]. This value is proportional to the measured *µ*_raf_ measured by HD-OCT. In the upper layer of normal skin at inner site of upper arm significant differences in *µ*_raf1_ according to intrinsic ageing could be measured by HD-OCT (unpublished data). In pre-menopausal and post-menopausal females the relative attenuation factor was found to be 0.0114/µm (±0.0012/µm) and 0.0375/µm (±0.0037/µm), respectively. It also appeared that *µ*_raf4_ at superficial reticular dermis was significantly higher in pre-menopausal women compared to post-menopausal women; 0.016/µm (±0.0012/µm) and 0.0094/µm (0.0005/µm), respectively (unpublished data). This suggested an intrinsic ageing-related decrease in anisotropy of scattering in upper layers and increase in anisotropy of scattering in lower layers.

In the epidermis, a significant difference (*p* < 0.001) in relative attenuation factor normalized to skin entrance signal could be observed among the three melanocytic subgroups. This factor is proportional to the *µ*′_s_ when a light source of 1300 nm is used [26, 29]. A strong reduction of *g* and consequently a robust increase in *µ*′_s_ could be noticed in BN compared to normal values [29]. Hence, a reduction of factor *µ*_raf1_ with increasing malignancy which could be observed in our study implied an important difference in anisotropy factor g among the three melanocytic subgroups. Lower g values (less than 0.8) corresponding with more isotropic scattering seemed to be characteristic for BN. In BN the *µ*′_s_ of the upper layer was approximately 3 times higher compared to normal skin. High g values (higher than 0.9) corresponding with strong anisotropic scattering seemed to be characteristic for melanoma. Forward scattering became more likely. In melanoma the upper layer *µ*′_s_ seemed to be 10 times lower than normal skin. Hence, the epidermis became much more translucent compared to BN and DN. As a consequence NIR light penetrated much deeper in melanoma than in DN or BN. This probably explains the observed differences in dermoscopic imaging.

Cellular and micro-architectural risk parameters used for diagnosing MM have been determined for HD-OCT such as large roundish pagetoid cells, atypical cell clusters at dermo-epidermal junction, totally disarranged epidermal/dermal pattern and large vertical icicle-shaped structures [9, 20]. With regard to the impact of scattering, alterations of distribution, size, density and orientation of scatterers with malignancy probably are of higher importance than the content of pigment itself because scattering is dependent on the refractive index mismatch between cellular components, extracellular matrix fibres and the extracellular fluid [51]. Dysplastic nevi are characterized by nuclear enlargement, slight irregularity and hyperchromasia, with clumping of chromatin and sometimes with prominent nucleoli. In melanoma besides nuclear atypia, nests and single melanocytes of variable sizes, shapes are present in the epidermis in a pagetoid pattern [16]. A decline of the relative attenuation factor demonstrated in our study suggesting an increase of g value is in line with former observations stating that the average scatterer size in melanoma is significantly larger than those of DN and BN [21, 39, 40].

Some important limitations need to be addressed. This study is a retrospective pilot study. However, this type of study is essential to gather information to design an appropriate (e.g. adequate sample size) prospective study. A second issue is the selected region of interest for optical analysis. This selection needs some experience with morphology analysis of HD-OCT images of melanocytic lesions. This fact questions to some extent the accuracy of HD-OCT optical analysis in the hands of non-experts. Last but not least, the effect of age and gender on optical properties was not taken into account.

In conclusion, HD-OCT seems to enable the rather unique combination of in vivo morphological analysis of cellular and 3-D micro-architectural structures with in vivo analysis of optical properties of tissue scatterers in melanocytic lesions. In vivo HD-OCT analysis of optical properties permits melanoma diagnosis with higher accuracy than in vivo HD-OCT analysis of morphology alone. The diagnostic performance of HD-OCT in MM should be further assessed in other clinical settings combining both types of analysis.
